# A Novel, Simple, and Low-Cost Approach for Machine Learning Screening of Kidney Cancer: An Eight-Indicator Blood Test Panel with Predictive Value for Early Diagnosis

**DOI:** 10.3390/curroncol29120715

**Published:** 2022-11-24

**Authors:** Haiyang Li, Fei Wang, Weini Huang

**Affiliations:** 1 Group of Cancer Evolution, School of Life Sciences, Sun Yat-sen University, Guangzhou 510275, China; 2 Department of Clinical Pharmacy, Dazhou Central Hospital, Dazhou 635000, China; 3 Department of Mathematics, Queen Mary University of London, London E1 4NS, UK

**Keywords:** early diagnosis, clear cell renal cell carcinoma, cancer screening, machine learning

## Abstract

Clear cell renal cell carcinoma (ccRCC) accounts for more than 90% of all renal cancers. The five-year survival rate of early-stage (TNM 1) ccRCC reaches 96%, while the advanced-stage (TNM 4) is only 23%. Therefore, early screening of patients with renal cancer is essential for the treatment of renal cancer and the long-term survival of patients. In this study, blood samples of patients were collected and a pre-defined set of blood indicators were measured. A random forest (RF) model was established to predict based on each indicator in the blood, and was trained with all relevant indicators for comprehensive predictions. In our study, we found that there was a high statistical significance (*p* < 0.001) for all indicators of healthy individuals and early cancer patients, except for uric acid (UA). At the same time, ccRCC also presented great differences in most blood indicators between males and females. In addition, patients with ccRCC had a higher probability of developing a low ratio of albumin (ALB) to globulin (GLB) (AGR < 1.2). Eight key indicators were used to classify and predict renal cell carcinoma. The area under the receiver operating characteristic (ROC) curve (AUC) of the eight-indicator model was as high as 0.932, the sensitivity was 88.2%, and the specificity was 86.3%, which are acceptable in many applications, thus realising early screening for renal cancer by blood indicators in a simple blood-draw physical examination. Furthermore, the composite indicator prediction method described in our study can be applied to other clinical conditions or diseases, where multiple blood indicators may be key to enhancing the diagnostic potential of screening strategies.

## 1. Introduction

In recent years, newly diagnosed cases of kidney cancer have increased year by year [1], with 90% of the cases being clear cell renal cell carcinoma (ccRCC) [2]. Previous studies have pointed out that the incidence rate of renal cancer in the elderly is much higher than in the younger population [3]. With the rapid explosion and growth of the global newborn population after the Second World War, all countries have faced the ageing population problem in the past decade [4,5]. As a senile disease, the incidence of renal cell carcinoma (RCC) is highest in people aged 60 to 70 [6]. The number of confirmed cases of renal cancer has recently increased yearly [7,8]. For ccRCC patients, the five-year survival rate of early-stage (TNM 1) ccRCC is 96%, while that of advanced-stage (TNM 4) patients is only 23% [9,10]. Consequently, as a potentially fatal disease, early screening is extremely important for the successful treatment of kidney cancer, and no low-cost screening strategies are currently available. This problem is especially important in low-income countries or countries with limited opportunities for physical examination and imaging of a large number of people. In these cases, early screening of renal cancer through simple blood tests may significantly impact life-saving strategies.

In these cases, early screening of renal cancer through simple blood tests may have a significant impact on a life-saving strategies [11]. From a clinical point of view, Urology is facing future challenges, such as implementing the wide use of biomarkers. Along with medical imaging, like ultrasound and computed tomography, biomarkers, if reliable, could be successfully used for early diagnosis of kidney cancer [12,13]. In most developing countries, people rarely have whole-body physical examinations [14]. Generally, patients will not go to the hospital for examination until they have symptoms, such as physical weakness and gross hematuria [15]. However, when kidney cancer has such symptoms, patients are basically in the advanced stage of cancer, which significantly increases the difficulty of treatment and results in a lower survival rate [16]. Therefore, simple and efficient means of early cancer screening can effectively assist in cancer diagnosis. Previous studies have pointed out that cancer patients’ physical function and blood biochemical indicators will change significantly compared with healthy people [17]. The possible clinical utility of biomarkers in urology has been investigated and well-reviewed in Mancini et al. [11]. For renal cancer, He et al. [18] investigated the ability of preoperative serum albumin (ALB) to globulin (GLB) ratio (AGR), which predicts the long-term mortality of RCC patients, and proved AGR is an inexpensive survival predictor to be considered for routine clinical use. Shah et al. [19] proved that the level of haemoglobin (HB) and the survival rate of renal cell carcinoma are significantly related. Checheriţă et al. [20], Lazich and Bakris [21] pointed out that blood potassium (K${}^{+}$) imbalance is widespread in patients with kidney disease, especially in patients with the renal tubular disease or reduced glomerular filtration rate. Many studies have proved that blood urea nitrogen (BUN), serum creatinine concentration (CREA), and uric acid (UA) are important indicators of kidney function [22,23,24]. In addition, a high level of high-sensitivity C-reactive protein (hs-CRP) in the blood is one of the reasons for the shortened survival time of patients with chronic kidney diseases and cancers [25,26]. All the above-mentioned studies propose some blood indicators that show different patterns of expression in patients with kidney cancer, detectable with a simple blood test. The aim of our study was to combine these indicators and utilize them as an eight-indicator panel, with potential predictive value for diagnosis of kidney cancer in the general population.

**Figure 1:**
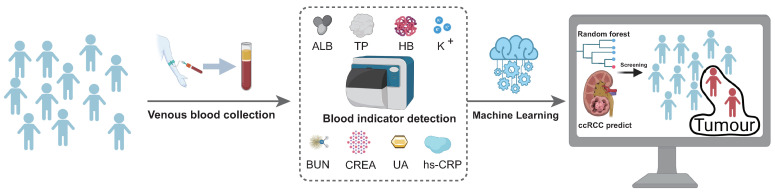
The scheme of early screening for kidney cancer and research protocol. We collected data from healthy people and patients diagnosed with ccRCC. The blood samples were measured by biochemical instruments for eight main indicators: ALB, TP, HB, K${}^{+}$, BUN, CREA, UA and hs-CRP. After the data were measured, the indicators were introduced into the RF model for training. Finally, the prediction model’s performance was tested to achieve accurate classification and prediction performance.

We collected peripheral blood samples from people diagnosed with early-stage ccRCC (TNM 1) and advanced-stage ccRCC (TNM 4). After that, we measured the indicators of peripheral blood and compared them with the indicators of blood samples collected from healthy people. Meanwhile, we analysed the correlation and differences between patient’s indicators. Then, the diagnosis and prediction of ccRCC were performed using a naive Bayesian model (single blood indicator) and a random forest model (mixed blood indicators). As shown in Figure 1, the random forest prediction model was trained to test the prediction performance of each indicator in the blood. All ccRCC-related peripheral blood indicators were combined for comprehensive prediction. The prediction differences of early and late indicators are compared simultaneously to improve the accuracy and prediction performance of the model. Early screening for renal cancer by simple test blood indicators is finally realised.

## 2. Materials and Methods

### 2.1. Patients and Samples

In this study, we collected 743 blood samples from patients with, renal cancer from September 2017 to September 2022 in Dazhou Central Hospital, including 505 blood samples from patients with early-stage ccRCC (TMN 1) and 237 blood samples from patients with advanced ccRCC (TMN 4). The age of cancer patients is mainly around 57 years old, and the interquartile range is 47–65 years old. Therefore, we randomly collected samples from 500 patients aged 50–65 as the control set, with an average of 56 years old. We collected venous blood samples from patients with medically and radio-logically confirmed ccRCC, using heparin as an anticoagulant. We centrifuged the collected sample at 4 ${}^{{}^{\circ}}$C at 3000 rpm for 30 min and separated the blood cells to prepare for the determination of blood indicators. Patient ALB, total protein (TP), HB, renal function indicators (BUN, CREA and UA), and inflammation indicators hs-CRP in blood samples were obtained and collected by the automatic biochemical analyser LABOSPECT 006 (Hitachi, Ltd., Tokyo, Japan) and Coulter Ac•T 5diff AL (Autoloader) Hematology Analyser (Beckman Coulter, Ltd., Indianapolis, IN, USA) using a MedicalSystem test kit (MedicalSystem Biotechnology Co., Ltd., Ningbo, China). We confirm that this study was conducted in accordance with the Declaration of Helsinki, and was approved by the ethics committee of Dazhou Central Hospital (protocol code 2022(052)).

### 2.2. Statistical Analyses

The clinical report was completed with the “gtsummary” package [27] in R language, in which the number and percentage of male and female populations are expressed. In contrast, age and other biochemical indicators are displayed by mean and interquartile range (IQR). The different analyses between biochemical indicators were carried out with the “limma” tool package (version 3.52; https://bioconductor.org/packages/limma (accessed on 10 October 2022)) developed by Ritchie et al. [28]. Continuous variables are presented as mean ± SD, and categorical variables are expressed as a number (percentage) or visualisation through R studio and Python. For correlation coefficient, 1 means positive correlation and −1 means negative correlation, which can measure the strength of the variable relationship. The closer to 0 correlation, the weaker the correlation. All the indicators were analysed and calculated. The following is the formula for calculating the correlation coefficient:

(1) $$Corr=\frac{\sum \left(x_{i}-\bar{x}\right)\left(y_{i}-\bar{y}\right)}{\sqrt{\sum {\left(x_{i}-\bar{x}\right)}^{2}\sum {\left(y_{i}-\bar{y}\right)}^{2}}}$$

where Corr is the correlation coefficient, $x_{i}$ is the values of the *x*-variable in a sample, $\bar{x}$ is the mean of the values of the *x*-variable, $y_{i}$ is the values of the *y*-variable in a sample, and $\bar{y}$ is the mean of the values of the *y*-variable.

For the significance analysis of statistical differences, we consider an observed test-statistic *t* from unknown distribution *T*. Then, the *p*-value *p* is what the prior probability would be of observing a test-statistic value at least as “extreme” as *t* if null hypothesis $H_{0}$ were true [29]. Therefore, the calculation of *p* is as follows:

(2) $$p=\mathrm{Pr}\left(\left|T\right|\geq \left|t\right||H_{0}\right)$$

In statistics, naive Bayes classifiers are a class of simple probabilistic classifiers based on the application of Bayes’ theorem and the assumption of strong (naive) independence between features [30,31]. The conditional probability model probability can be decomposed as:

(3) $$\;\mathrm{posterior}\;=\frac{\;\mathrm{prior}\;\times \;\mathrm{likelihood}\;}{\;\mathrm{evidence}\;}\Rightarrow p\left(C_{k}|x\right)=\frac{p\left(C_{k}\right)p\left(x|C_{k}\right)}{p(x)}$$

where $C_{k}$ is the class variable, corresponding with vector **x**.

In model research, the ROC curve is often used to evaluate a model’s effectiveness and test whether the model has practical value [32,33]. The “pROC” package (version 1.18; https://cran.r-project.org/web/packages/pROC/ (accessed on 10 October 2022)) visualizes the ROC curve and AUC of our model, where AUC is a critical index in ROC curve that tests whether positives are ranked higher than negatives. The AUC is equivalent to the Wilcoxon or Mann–Whitney U test [34] statistic, with the relation as follows:

(4) $$AUC\left(f\right)=\frac{\sum_{t_{0}\in U^{0}}\sum_{t_{1}\in U^{1}}1\left[f\left(t_{0}\right)<f\left(t_{1}\right)\right]}{\left|U^{0}\right|\cdot \left|U^{1}\right|}$$

where $1[f\left(t_{0}\right)<f\left(t_{1}\right)]$ denotes an indicator function that returns 1 if $f\left(t_{0}\right)<f(t_{1})$ and otherwise returns 0; $U^{0}$ is the set of negative examples, and $U^{1}$ is the set of positive examples. After ROC curve analysis of all blood biochemical indicators, renal function-related indicators with higher AUC indicators were selected for further analysis.

### 2.3. Modelling of Early Screening Models

We constructed a naive Bayesian classification model [30] to predict a single index and introduced a single biochemical indicator of healthy people and ccRCC patients into the model for training to obtain the ROC prediction performance curve and the corresponding AUC of each of the eight indicators. The random forest (RF) classifier [35] is an integrated machine learning method that is a collection of decision trees. The final decision of RF is to make a majority vote in all trees to produce a more accurate classification, and it has been widely used to solve classification difficulties. Compared with other popular classifiers [36], RF is recognised as a good classification method. Meanwhile, a naive Bayesian model is suitable for building and further analysing enormous data sets. This model is a straightforward compound classification method that can classify well even in complex situations. In this study, eight blood indicators were normalised, and the data were grouped by random sampling. We divided healthy individuals and ccRCC patients into training sets (80%) and verification sets (20%). The RF model is based on Python (version 3.9; https://www.Python.org (accessed on 10 October 2022)) and the “sklearn” library (version 1.1.2; https://scikit-learn.org/stable (accessed on 10 October 2022)). The GridSearchCV module was used to automatically adjust the parameters of the RF model with about 100 trees, each with eight randomly selected variables and a maximum tree depth of 50 to achieve the best results for the model. We collected the results and selected the model with the best performance while measuring the prediction accuracy on the test set. At the same time, the prediction accuracy was measured on the test set. Then, the model was optimised for the number of variables selected for each tree. In this process, the over-fitting of the RF model during parameter adjustment was prevented by cross-validation, so as to keep the stability and practicability of the model. Model performance evaluation is based on the ROC curve and corresponding AUC value.

### 2.4. Data Visualisation

Data visualisation and statistical analysis were both carried out using R (version 4.2.1, https://www.r-project.org/ (accessed on 10 October 2022)) and Python 3.9. The main plot and statistical significance was visualized using the “ggplot2” package (version 3.3.6; https://cran.r-project.org/web/packages/ggplot2 (accessed on 10 October 2022)) in R. The basic column chart and box diagram are drawn by the “matlibplot” python package (version 3.5; https://matplotlib.org/ (accessed on 10 October 2022)). The correlation coefficients between the data indices in the study were visualized by the “ggcorrplot” package (version 0.1.3; https://cran.r-project.org/web/packages/ggcorrplot/ (accessed on 10 October 2022)). A heat scatter was created using the “LSD” package (version 4.1-0; https://cran.r-project.org/web/packages/LSD (accessed on 10 October 2022)) in R. The indicator differences between male and female medical records of different cancers were visually compared by the “beanplot” package (version 1.3.1; https://cran.r-project.org/web/packages/beanplot/ (accessed on 10 October 2022)). The visualisation of the pair plot was performed through the “seaborn” python package (version 0.11.2; https://seaborn.pydata.org/ (accessed on 10 October 2022)). Otherwise, optimisation of colour and typesetting was completed with Adobe Illustrator (https://www.adobe.com (accessed on 10 October 2022)).

## 3. Results

### 3.1. The Demographic Characteristics of All Samples

**Figure 2:**
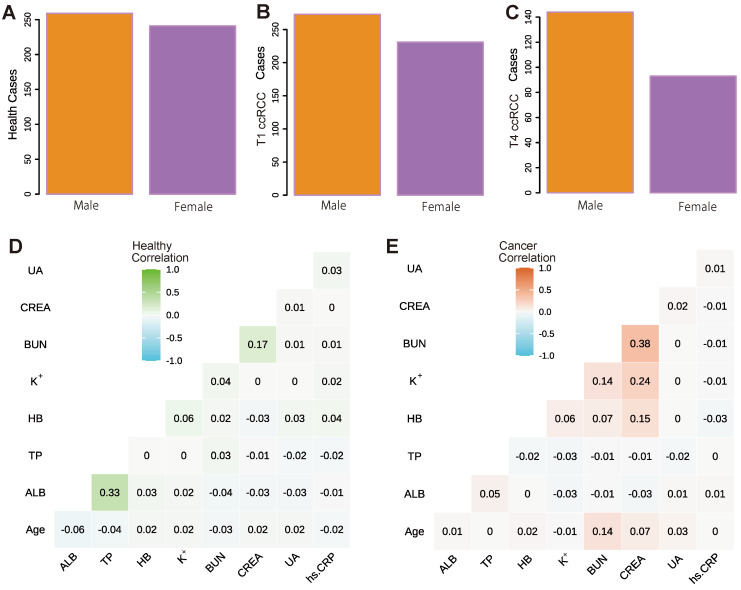
The distribution of male and female population and correlation analysis between blood indicators of healthy and cancer samples. (**A**) The distribution of men and women in the healthy population. (**B**) The distribution of men and women in the early-stage cancer population. (**C**) The male and female distribution in the advanced cancer population. (**D**) The correlation analysis results between the healthy population’s blood indicators. (**E**) The results of correlation analysis between blood indicators of the early-stage cancer population.

In this study, the ratio of males to females in the healthy population is approximately 1:1. In early-stage ccRCC, the ratio of males is 273:505, accounting for 54.1%, which is slightly higher than that of females, while in advanced ccRCC, the ratio of males is much higher than that of female, accounting for 60.8%. The detailed distribution is shown in Figure 2A–C. The average age of healthy people is 56 years old and that of people with cancer is 57 years old, but the age fluctuation range is slightly different. The age range of early-stage ccRCC is 47–65 years old, while that of advanced cancer is 54–65 years old. This also shows that advanced cancer is more likely to occur in older people.

Furthermore, we analysed the correlation between eight key biochemical indicators in healthy people and early-stage ccRCC patients. In the results, only ALB and TP have a strong correlation in healthy people, because TP is the sum of ALB and GLB and the value of TP is partly determined by ALB. Notably, BUN and CREA also have a strong positive correlation (Figure 2D). In fact, BUN can reflect the kidney condition like CREA can, because BUN is the same as blood creatinine. It is one of the ultimate protein metabolic products, mainly through the filtering function of glomerular balls to discharge in vitro. Therefore, BUN and CREA show a strong correlation.

In the cancer population, the renal function indicators (BUN, CREA, and UA) are all positively correlated with the patient’s age, indicating that the renal function indicators also increase with the increase of age. As shown in Figure 2E, BUN and CREA have the same trend in the healthy population, showing a strong correlation. The difference is that the correlation between BUN and CREA in the healthy population is lower than that between ALB and TP. In comparison, the correlation between BUN and CREA in cancer patients is far more significant than that between ALB and TP. In addition, there is a positive correlation between BUN, CREA, K${}^{+}$, and HB indicators, among which the correlation between CREA and BUN, and CREA and HB are the most obvious. Clinically, urea nitrogen and creatinine in the blood are products of protein metabolism; therefore, both have a positive correlation with protein (HB) rising.

### 3.2. Analysis of Blood Biochemical Indicators

**Figure 3:**
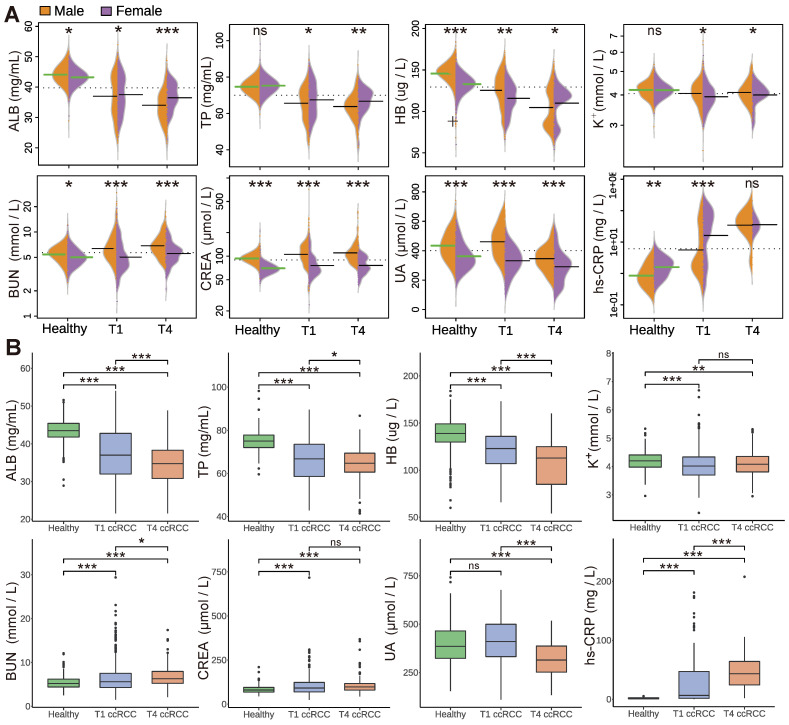
The distribution of the significant difference between indicators. (**A**) The significant difference between male and female indicators in the healthy populations and cancer patients. (**B**) The statistical significance analysis of ccRCC and healthy people. The statistical significance analysis of indicators with protein, renal function, inflammation, etc. (*** means “*p* < 0.001 statistically significant”; ** means “*p* < 0.01 is statistically significant”; * means “*p* < 0.05 is statistically significant”; insignificance is denoted by ns).

After comparing the correlation of blood indicators of patients in each group, we also analysed eight indicators previously showed to be involved in kidney diseases. Due to the differences between men and women in constitution, endocrinology, and normal range of blood indicators [37], we compared the statistical significance between men and women in the analysis process, as shown in Figure 3A. *** means *p* < 0.001 statistically significant; ** means *p* < 0.01 is statistically significant; * means *p* < 0.05 is statistically significant; insignificance is denoted by ns. In terms of protein indicator results, ALB and TP indicators between healthy men and women were not significantly different. ALB and TP of early-stage ccRCC began to show statistical significance (*p* < 0.05), while the male and female patients with advanced ccRCC (TNM 4) showed high statistical significance (*p* < 0.01). On the other hand, there is a significant difference in haemoglobin between healthy men and women. With the development of cancer, This may be because more advanced cancer consumes a large amount of HB. Therefore, more advanced tumours better balance the differences between men and women. This result is also reflected in hs-CRP, and there is no significant difference between men and women in advanced ccRCC patients.

As a result, for early diagnosis, women could benefit more from the panel proposed in this study, while in case of advanced tumours, women and men are equally well performing regarding the diagnostic ability of the panel. However, there is a statistical significance (*p* < 0.001) between male and female patients in renal function indicators, and there is a statistical significance (*p* < 0.05) in both healthy people and cancer patients. Another key point is that in the analysis of male and female indicators, the values of ALB, TP, HB and K${}^{+}$ of healthy people are above the average (dashed line in Figure 3A). The indicators of cancer patients decreased significantly compared with those of healthy people. On the contrary, renal function indicators (BUN, CREA, and UA) in the healthy population are significantly lower than the mean, while those in cancer patients are significantly higher. Moreover, as the cancer cycle increases, the inflammatory indicator hs-CRP also shows the same situation, indicating that renal cancer causes the rise of renal function indicators and increases inflammatory factors in patients. By combining the above parameters with our model, the published data can be machine-learned to improve the accuracy of prediction.

As well as the analysis of male and female blood indicators, we compared the significance of differences between indicators in different groups of samples from healthy and cancer populations. This allows us to identify specific differences between healthy people and people with cancer. The protein groups of early- and late-stage ccRCC showed statistical significance. The protein indicators of cancer patients were significantly lower than those of the healthy population. In addition, advanced cancer consumed relatively more endogenous proteins. Therefore, ALB, TP, and HB indicators showed a significant decline. For indicators K${}^{+}$ and CREA, there is no significant difference, indicating that more advanced kidney cancer does not affect the indicators of K${}^{+}$ and CREA. TP and BUN begin to show statistical significance (*p* < 0.05), while other indicators show extreme statistical significance (*p* < 0.001). Previous studies have pointed out that early-stage renal cancer does not affect renal function, and our results show that UA also shows consistent characteristics. In addition, ha-CRP mainly shows inflammation in patients. It can be clearly found from the indicator comparison that the inflammation indicator in cancer patients is much higher than that in the healthy population, and the hs-CRP indicator of advanced TNM 4 patients is much higher than that of early-stage cancer patients.

### 3.3. The Clinical Significance of the Blood Indicator Ratio

**Figure 4:**
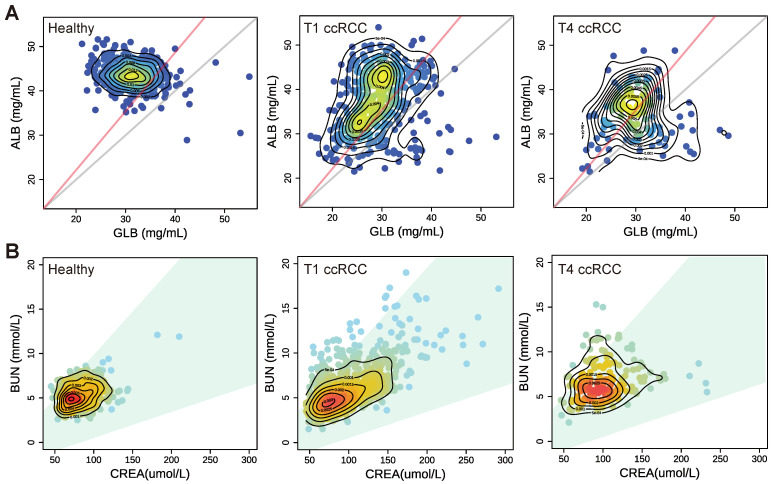
The distribution of AGR and BCR in healthy and cancer populations. (**A**) The distribution of AGR in the healthy population and ccRCC patients (red line is AGR = 1.2; grey line is AGR = 1). (**B**) The CREA: BUN ratio distribution in the healthy population and ccRCC patients (the green area is the BCR normal range 20–100).

We systematically analysed eight related indicators of peripheral blood measurement, where TP level mainly reflects the loss of protein caused by renal lesion and serum ALB is also be greatly reduced in cancer patients. GLB is closely related to human immunity. When the human body is invaded by viruses or cancer cells, GLB will rapidly increase [38]. As we know, the total protein is the sum of albumin and globulin, so ALB/GLB ratio (AGR) is one of the key indicators in the combination of indicators for screening cancer. Detection of abnormalities can be made earlier by examining the AGR so that patients can prevent kidney complications and cut off the development process of renal cancer earlier. In particular, the AGR is often used as a critical nutritional reference value before clinical operation. In general, an albumin/globulin ratio between 1.2 and 2.5 is considered normal, although this may vary depending on the laboratory tests [39]. Human blood usually contains a little more albumin than globulin, which is why the normal ratio is slightly higher than 1. An AGR lower than 1.2 indicates that patients have severe nutritional problems with protein. As shown in Figure 4A, the red line indicates the demarcation line of AGR of 1.2, and the grey line indicates the demarcation line of the AGR ratio of 1:1, which also indicates that protein deficiency is severe. The results showed that the value for healthy people was above 1, while the value for patients with early T1 ccRCC was below 1, suggesting a serious problem. Moreover, the warning line of 1.2 passes through the high-density area of the cancer population. Half of the patients with advanced T4 are below the red line, which also indicates that malignant renal cancer is negatively correlated with AGR. Suh et al. [40] have also demonstrated that a lower AGR is associated with a higher risk of death in cancer patients.

In renal function indicators, CREA and BUN can reflect the degree damage to glomerular filtration function to some extent. BUN is also affected by extra-renal factors, such as a high-protein diets, gastrointestinal bleeding, dehydration, and high catabolism, which can cause BUN to increase. CREA is more accurate than BUN because it mainly depends on glomerular filtration capacity when exogenous creatinine intake is stable, and creatinine production in vivo is constant [41]. Therefore, the observation of the BUN/CREA ratio (BCR) in serum has certain clinical significance. Furthermore, the level of BCR reflects the quality of renal function. Figure 4B shows the distribution of BUN and CREA in healthy people and cancer patients. The green area in the figure shows the range of normal values (20–100) of BCR. Healthy people are basically in the normal range. More patients with cancer are in the normal range, and the proportion of ccRCC in TNM 4 is higher outside the range than that in the patient population with TNM 1. Its significance is mainly that when BUN or creatinine CREA values are increased, it can be used as the judgment of the difference between the causes of renal or pre-renal (extra-renal) cancer. A BCR less than 20 indicates a high risk of renal disease [42]. The influence of the tumour increases the urea nitrogen value, so cancer may lead to a BCR greater than 100.

**Figure 5:**
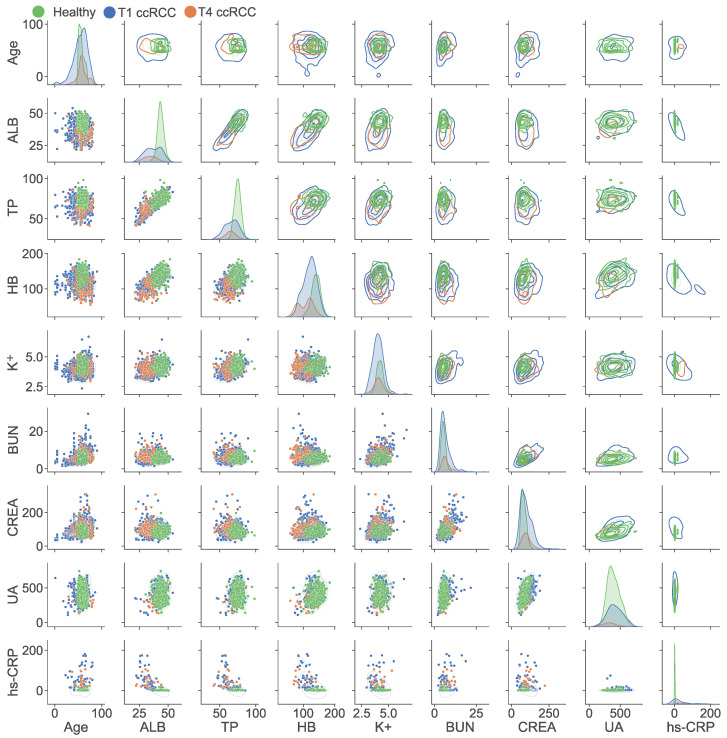
The paired relationship between biochemical indicators with ccRCC patients and healthy population. Correlation analysis of pairwise parameter combinations of the eight indicators was conducted to visualise the distribution differences between cancer patients and healthy people.

Many correlations and interactions between indicators were challenging to mine individually. Therefore, we performed the pairwise comparative analysis on patient age and the distribution of the eight key indicators, and conducted an in-depth analysis of the indicators through the scatter diagram and density diagram. The upper part of Figure 5 shows the density distribution of the 8 indicators. The scatter distribution of samples between groups is shown in the lower part of Figure 5, where the distribution of each patient and the indicator relationship between groups can be seen. In the figure, green indicates the healthy population, blue indicates early-stage ccRCC, and orange indicates advanced ccRCC. Among cancer patients, the age distribution curve ranges widely, but the peak is concentrated around the age of 60. In addition to the UA indicator of the healthy population basically covering cancer patients, the healthy population in other indicators was included in the scope of cancer patients.

### 3.4. Performance Test of Cancer Prediction Model

**Figure 6:**
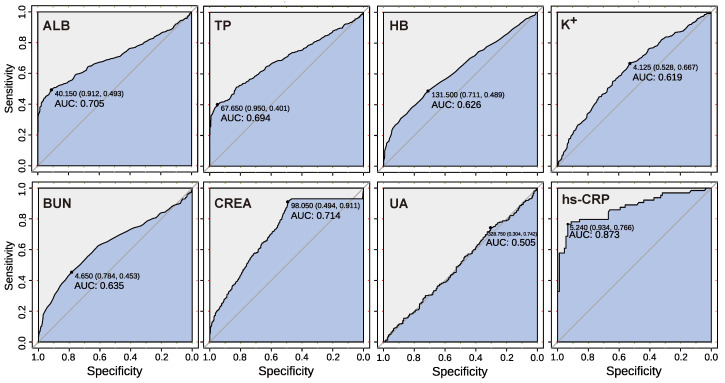
The ROC curve of the single indicator prediction model. The predictive effect of eight single indicator prediction models can be quantified by AUC to evaluate the efficiency of the model prediction.

An ROC curve is a graphical technique that is often applied to visualise classifier performance. For the single indicator prediction model, ROC was used to determine the prediction effect of the model. In this study, we tested the classification prediction performance of single indicator data of healthy population and early-stage ccRCC patients using the prediction model, as shown in Figure 6. The results showed the prediction performance of all eight key indicators.

**Figure 7:**
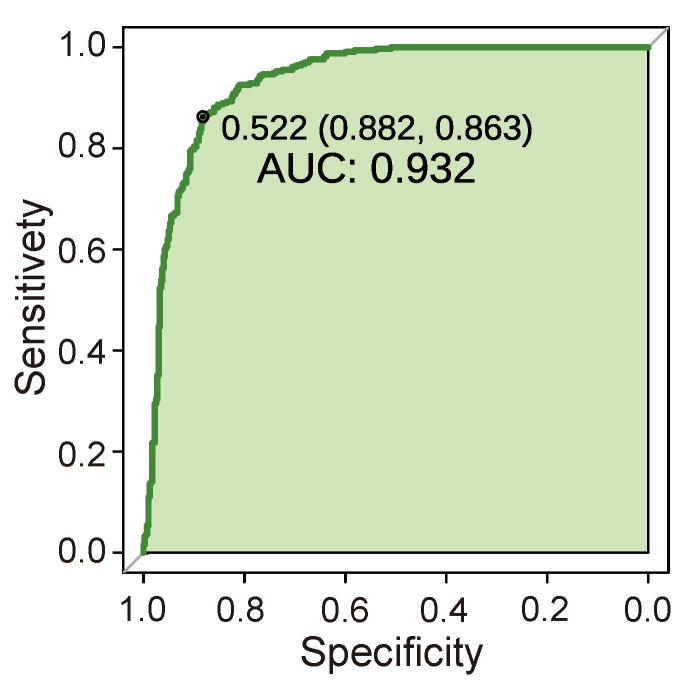
The ROC curve of the RF prediction model. The predictive effect of the eight-indicator prediction model can be quantified by AUC to evaluate the efficiency of the model prediction.

Except for the fact that UA in the renal function indicators had little predictive performance, all other indicators had high AUC values, among which HB, K${}^{+}$, and BUN had generally good performance, with AUCs around 0.6. The indicators of ALB, TP, and CREA had good performance, with AUCs above 0.7. Among them, ALB and TP have reasonable specificity above 90%, while CREA has poor specificity, but a high sensitivity of 91.1%. In addition, among the single-indicator prediction models, the inflammatory indicator hs-CRP performed best. The AUC of the model was up to 87.3%, the specificity was up to 93.4%, and the sensitivity was also 76.6%. Finally, the results show that the single-indicator prediction models of the ALB, TP, CREA, and hs-CRP indicators have good prediction effects. We trained the RF model with data sets of healthy people and cancer patients. The AUC of the RF prediction model was verified by combining eight key renal cancer prediction indicators. Our model has good prediction performance, with an AUC of 0.932 (Figure 7), a sensitivity of 88.2%, and a specificity of 86.3%. These findings indicate that random forest-based prediction can provide a satisfactory alternative biopsy method for ccRCC patients. In particular, high specificity and sensitivity may make our method useful for the early screening of renal cancer.

## 4. Discussion

In this study, the peripheral blood of early-stage ccRCC (TNM 1) was collected, and healthy people and advanced ccRCC (TNM 4) blood samples were used as control groups. After measuring the indicators of peripheral blood samples, the correlation between the indicators of early-stage ccRCC patients and the differences of healthy people were analysed. The results showed that cancer patients had lower protein levels, while renal function and inflammation indicators were higher than healthy ones. In addition, there was a high positive correlation between renal function indicators and a large difference between male and female indicators. However, more advanced tumours narrowed the difference between male and female indicators. Analysis of the blood indicators of healthy people and cancer patients can preliminarily classify the patients, with samples whose indicators exceed the standard have a higher risk of cancer. Correspondingly, we applied a naive Bayesian model (single blood index) and a random forest model (mixed blood index) to diagnose and predict ccRCC. The results are shown in Figure 6. A random forest prediction model was trained to test the prediction performance of each indicator in the blood. All the peripheral blood indicators related to ccRCC were combined for comprehensive prediction, and a good prediction performance was obtained. The AUC of the RF prediction model was verified at 0.932 (Figure 7). The sensitivity and specificity of the RF model in the validation queue of eight indicators were 88.2% and 86.3%, respectively. In summary, the early screening of renal cancer was realised by simple blood tests.

Gender differences exist in the incidence of kidney cancers, hormones, blood indicators, and the prognosis of the disease [43,44]. Nevertheless, clinical trials and studies of renal cancer are always unbalanced in terms of gender. Most data on gender differences in kidney cancer comes from studies published in developed countries [44]. In this study, we analysed the differences between male and female indicators in detail through the combination of blood indicators and models to improve the diagnostic potential of screening in women. Therefore, our model, when applied in countries where women have limited access to healthcare, can enhance the screening possibilities for this specific portion of the population, so that they could be diagnosed at an early stage. At more advanced stages, the differences between men and women tend to be less pronounced. Cancer patients have lower protein levels than healthy people [45], Furthermore, the inflammatory indicator hs-CRP has been proven to increase [46]. The changes in indicators in ccRCC patients in our results (Figure 3) are consistent with the trends in previous studies. Yim et al. [47] proved that the renal function of patients with early-stage renal cancer is basically unaffected, and uric acid tends to be low with tumour development. Norberg et al. [48] also pointed out that renal cancer increases levels of creatinine, urea nitrogen, and other indicators with the development of tumours. This kind of situation also existed in patients with renal cancer in our research results (Figure 3B). High CREA and BUN levels in blood represent the decrease of glomerular filtration rate and renal detoxification function. At this time, the kidney function of patients with nephropathy begins to be damaged and enters the stage of renal insufficiency. With the continuous increase of CREA and BUN in blood, the degree of renal function damage will become much more serious. However, the decrease of UA may be due to the gradual weakening of the metabolism and intracellular enzyme activity, resulting in the weakening of biochemical reactions of UA metabolism. Previous studies have used serum albumin/globulin ratio (AGR) to predict long-term mortality [18,49] and predict the prognostic effect of screening by the same method [50] in ccRCC. A low albumin/globulin ratio may put patients at risk for cancer. In an observational study of nearly 27,000 people, participants with an AGR below 1.2 had an increased risk of cancer [40], even if they were otherwise healthy. In addition to being associated with risk of cancer, AGR may also indicate the extent to which cancer patients respond to treatment [51]. Moreover, He et al. [52] used 13,890 patients from 24 articles for an analysis of overall survival (OS); compared to lower AGR patients, higher AGR patients had better OS. Our results also show that with the development of cancer, more populations deviate from normal values of AGR and BCR (Figure 4). These results also prove the levels of AGR and BCR are greatly affected by cancer.

Prediction of the clinical behaviour of cancer by artificial intelligence is a hot topic in contemporary research [53]. The RF model has been previously used in disease and cancer prediction, and most cancer prediction models test model performance by the AUC value of the ROC curve. Wang et al. [54] used four genes to predict ccRCC to guide immunotherapy and their RF model’s AUC was 0.78. Erdim et al. [55] constructed a random forest model with an AUC as high as 0.916; this model was recognised as a suitable method to distinguish benign and solid renal tumours. Our eight-indicator RF prediction model shows good predictive performance, with an AUC reaching 0.932. Therefore, the RF model in this study can be used as an effective tool for the early screening of renal cancer.

Our study has some limitations: the data comes from a single center, the patients’ number is limited, and all the patients belong to the same race. Moreover, the statistical data is relatively simple. We can improve the statistical power of the study in the next future, by adding more data and more variability, and by introducing more research on the patients’ follow-up.

## 5. Conclusions

This study focused on developing a low-cost, patient-friendly, and effective strategy for kidney cancer screening. A potential clinically useful combination of eight biomarkers, all “renal” indicators detectable with a simple blood analysis, were studied through an RF prediction model, which achieved a sensitivity of 88%, a specificity of 86.3%, and an AUC of 0.932.

These findings indicate that random forest-based prediction can be used as a reliable method for screening with high specificity and sensitivity, potentially acting as a low-cost liquid biopsy for ccRCC patients. This strategy, useful for early screening worldwide, could become particularly crucial in the large part of the world where more expensive screening programs are not possible and portions of the population are excluded from physical medical examinations or imaging.

Additionally, the comprehensive indicator prediction method applied in our research can also be used to predict the risk of harbouring other diseases whose presence has been correlated to alterations in specific blood indicators, as is the case in kidney cancer.

## Data Availability

The original contributions presented in this study are included in the article. Further inquiries can be directed to the corresponding author.
